# Intracellular Thermal Probing Using Aggregated Fluorescent Nanodiamonds

**DOI:** 10.1002/advs.202103354

**Published:** 2021-11-23

**Authors:** Tianli Wu, Xixi Chen, Zhiyong Gong, Jiahao Yan, Jinghui Guo, Yao Zhang, Yuchao Li, Baojun Li

**Affiliations:** ^1^ Institute of Nanophotonics Jinan University Guangzhou 511443 China; ^2^ Department of Physiology, School of Medicine Jinan University Guangzhou 510632 China

**Keywords:** cell thermometry, fluorescent nanodiamonds, intracellular detection, nanoprobes, optical trapping and manipulation

## Abstract

Intracellular thermometry provides important information about the physiological activity of single cells and has been implemented using diverse temperature‐sensitive materials as nanoprobes. However, measuring the temperature of specific organelles or subcellular structures is challenging because it requires precise positioning of the nanoprobes. Here, it is shown that dispersed fluorescent nanodiamonds (FNDs) endocytosed in living cells can be aggregated into microspheres using optical forces and used as intracellular temperature probes. The aggregation of the FNDs and electromagnetic resonance between individual nanodiamonds in the microspheres lead to a sevenfold intensity enhancement of 546‐nm laser excitation. With the assistance of a scanning optical tweezing system, the FND microspheres can be precisely patterned and positioned within the cells. By measuring the fluorescence spectra of the microspheres, the temperatures at different locations within the cells are detected. The method provides an approach to the constructing and positioning of nanoprobes in an intracellular manner, which has potential applications in high‐precision and flexible single‐cell analysis.

## Graphical abstract

Endocytosed fluorescent nanodiamonds (FNDs) dispersed in the cells are aggregated and positioned with high precision and flexibility by optical force. The aggregation of the FNDs and electromagnetic resonance between individual FNDs in the microspheres lead to a sevenfold intensity enhancement of 546‐nm laser excitation. The aggregated microspheres can be used as intracellular thermal probes for temperature detection of desired locations of the cells.

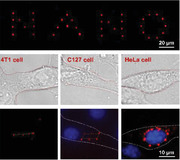

## 1 Introduction

Temperature is a fundamental indicator of cell function because all physiological activities involve exothermal or endothermal processes in specific organelles.^[^ ^1^, ^2^ ^]^ Detection of intracellular temperature has therefore become important for studying cell behavior and interactions.^[^ ^3^, ^4^, ^5^, ^6^ ^]^ To implement intracellular thermometry, diverse materials with temperature‐sensitive fluorescence intensities or spectra have been used, such as quantum dots (QDs),^[^ ^7^, ^8^, ^9^ ^]^ upconverting nanoparticles (UCNPs), nanogels,^[^ ^10^ ^]^ and thermosensitive dyes.^[^ ^11^ ^]^ Because QDs are constructed from semiconductor materials, they need to be coated with silicon dioxide to be biocompatible.^[^ ^9^ ^]^ To obtain higher biocompatibility, UCPNs are often used in deep tissues,^[^ ^12^ ^]^ but their anti‐Stokes luminescence has a quantum efficiency of only 0.01–5%. Organic dyes can be easily incorporated into living cells, but these fluorophores have low photostability and considerable dependence on the local environment (e.g., pH, pressure, and ion concentration).^[^ ^13^ ^]^ Nanogels can measure the average temperature of a whole cell, but challenges in manually controlling their distribution and position make it difficult to determine the intracellular temperature at specific locations.^[^ ^14^ ^]^ Therefore, there has been a need to develop biocompatible nanothermometers with high photostability and controllability for intracellular thermometry.

Fluorescent nanodiamonds (FNDs) containing nitrogen‐vacancy (NV^−^) centers^[^ ^15^ ^]^ are appealing fluorescent markers because of their inherent biocompatibility,^[^ ^16^, ^17^ ^]^ high photostability,^[^ ^18^, ^19^, ^20^ ^]^ sensitive temperature detection,^[^ ^21^, ^22^, ^23^, ^24^ ^]^ and suitability for intracellular use.^[^ ^25^ ^]^ With these features, FNDs have become a prominent competitor in the temperature‐sensitive nanomaterials for intracellular thermometry (see Table S1, Supporting Information, for a detailed comparison). To improve their performance, an efficient method of assembling the FNDs is required because their fluorescence intensity can be markedly increased by accumulating the diffuse nanoparticles. Among the techniques that have been developed, self‐assembly is a simple and low‐cost method to autonomously assemble particles;^[^ ^26^, ^27^, ^28^ ^]^ however, its application is restricted to thermodynamically stable colloidal structures. Acoustic‐ and magnetic‐assisted methods have also been developed to create freestanding nano‐assemblies of chains and films,^[^ ^29^, ^30^, ^31^, ^32^ ^]^ but the method is generally implemented on a large scale and rather than in single cells. In comparison, optical assembly enables easier fabrication and more flexible manipulation of nano‐ and microstructures,^[^ ^33^, ^34^, ^35^, ^36^, ^37^, ^38^ ^]^ especially in three dimensions. A distinctive method of optical assembly uses a photothermal effect (opto‐thermophoretic tweezers) to assemble 500‐nm polystyrene particles into 2D and 3D hydrogel structures by immobilizing them on a substrate using UV crosslinking.^[^ ^39^, ^40^, ^41^ ^]^ Despite being a simple and reliable way to build superstructures using low optical power, thermal conversion requires a gold film, which limits its application to single cells. Nanoparticles with chemical or protein modifications enable imaging with a high spatial resolution,^[^ ^42^, ^43^, ^44^, ^45^, ^46^ ^]^ but their assembly and detection can only be implemented at specific locations because organelle binding is required.

To implement FND‐based intracellular thermometry at any desired locations or organelles with high precision and flexibility, here we describe a method of aggregating FNDs into microspheres and positioning them at desired locations in living cells using optical tweezers. We demonstrate that the enhanced fluorescence intensity resulting from aggregation and interaction of FNDs can be used as highly sensitive intracellular thermal probe.

## 2 Results

### 2.1 Experiment Design and Materials Characterization

**Figure 1:**
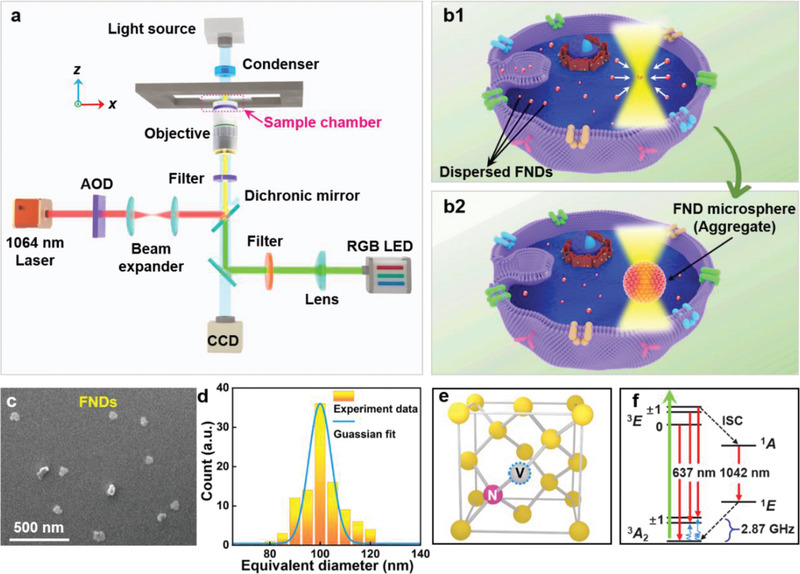
a) Experimental setup. b) Schematic of the intracellular aggregation of FNDs. The dispersed FNDs were endocytosed by living cells and then attracted by an optical trap (b1). More FNDs were gradually aggregated into a microsphere by the continuous action of the trapping laser (b2). c) Scanning electron microscope (SEM) image of the FNDs used in the experiments. d) Size distribution of the FNDs. e) Atomic structure and f) energy level diagram of the NV^−^ center in the FND. The structure contains a carbon vacancy (gray sphere with blue dashed circle) paired with a substitutional nitrogen impurity (pink sphere). The green and red arrows and the blue sinusoid in (f) denote optical excitation, fluorescence emission, and microwave excitation, respectively.

**Figure** **1**a presents a schematic of the experimental setup comprising a scanning optical tweezing system (SOTS), a 60× water‐immersion inverted objective (numerical aperture: 1.0), and a trapping laser beam (wavelength: 1064 nm) focused on the sample chamber to aggregate the FNDs (see the Experimental Section and Figure S1 , Supporting Information, for further details). The aggregated FND microspheres were excited at 546 nm by an RGB LED (see the Experimental Section for further details). The intracellular aggregation is schematically shown in Figure 1b. The FNDs were endocytosed and dispersed throughout the cell. The SOTS then attracted and optically trapped the FNDs within the cell (Figure 1b). During continuous application of the trapping laser, FNDs gradually aggregated to form a microsphere (Figure 1b2). The size of the aggregated FND microsphere could be adjusted by varying the power of the trapping laser, as to be described in later sections. The FNDs used in this work possessed a relatively uniform size (Figure 1c) with an equivalent diameter of 100 ± 30 nm (Figure 1d) and contained a high‐density ensemble of fluorescent NV^−^ centers (≈300 centers per particle). In the sp^3^‐hybridized FND lattice structure, the NV^−^ center is a point defect at which two adjacent carbon atoms are replaced by a substitutional nitrogen atom and a vacancy (Figure 1e).^[^ ^47^ ^]^ The NV^−^ center absorbs 550‐nm light during the electronic transition between the ground (^3^A_2_) and optically excited (^3^E) triplet states (Figure 1f) and has an emission at 685 nm with a high quantum yield. The fluorescence of the FNDs is completely stable, neither photobleaching nor photoblinking. The NV^−^ centers were selected to construct the nanothermometer because they undergo thermal shifts of both their resonant spin transition at 2.87 GHz and optical transition with zero phonon line (ZPL) at 637 nm.^[^ ^22^ ^]^

### 2.2 Formation and Patterning of FND Microspheres

**Figure 2:**
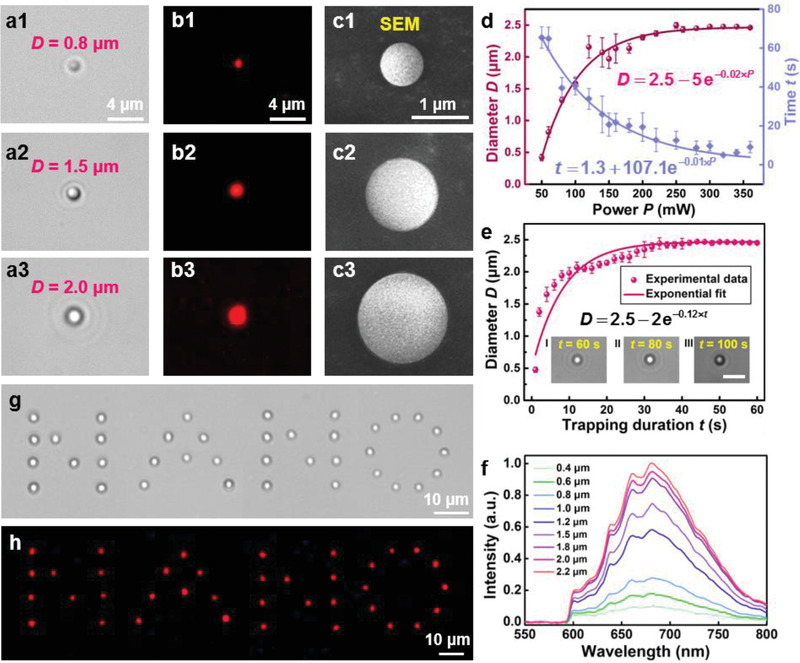
a) Aggregated FND microspheres with diameters reaching 0.8 (a1), 1.5 (a2), and 2.0 µm (a3) by applying a trapping laser (power: 60 mW) for different times. b) Fluorescence images of the aggregated FND microspheres at a 546‐nm excitation light. c) SEM images of the aggregated FND microspheres corresponding to (a1–a3). d) FND microsphere diameters and stabilization time as functions of the trapping power. Error bars represent standard deviation of five representative measurements. e) FND microsphere diameter as a function of the trapping duration. Error bars represent the standard deviation of five representative measurements at the same trapping power. The insets show the aggregated FND microspheres with the durations of 60 (I), 80 (II), and 100 s (III), respectively. Scale bar: 5 µm. f) Normalized fluorescence intensity of the aggregated FND microspheres with different diameters. g) Patterning of the word “NANO” on the glass substrate using a customized scanning optical trap array. h) Fluorescence image of the pattern “NANO” at a 546‐nm excitation light.

To investigate the performance of the proposed aggregation method, an optical trap created by the SOTS was first applied to the FND solution to form microsphere (**Figure** **2**a). The diameter of the aggregated microsphere reached 0.8 (Figure 2a1), 1.5 (Figure 2a2), and 2.0 µm (Figure 2a3) after applying the trapping laser (power: 60 mW) for 5, 17, and 30 s, respectively (see Figure S2 and Video S1, Supporting Information, for the detailed aggregation process). For optical trap durations longer than 100 s, the aggregated FND microsphere could maintain its shape and size even if the trapping laser was removed, a phenomenon attributed to electrostatic interactions between the FNDs^[^ ^48^ ^]^ (Figure S3, Supporting Information). An increase in ionic strength of the solution will therefore enhance these interactions and alter the formation and stabilization rates of the aggregates (see Section S2.4 and Figure S4, Supporting Information, for the aggregate formation at different salt concentrations). After 546‐nm LED excitation at a density of 33.40 ± 0.01 mW cm^−2^, fluorescence images of FND microspheres were obtained using a high‐definition color microscope camera (model: DS‐Fi3, Nikon, Japan) equipped with a 5.9‐megapixel CMOS image sensor (Figure 2b). The 8‐bit color depth of the camera enabled capture of fluorescence images of FNDs with a quantum efficiency of 65% at 546 nm. Scanning electron microscopy (SEM) images show that the FND microspheres had regular spherical shapes and smooth surfaces (Figure 2c) and their diameter ranged from 0.4 to 2.5 µm. Larger FND microspheres had greater roundness (see Section S2.5 and Figure S5, Supporting Information), and the minimum size of stable FND aggregates having a satisfactory roundness was 0.4 µm (Section S2.6 and Figure S6, Supporting Information). The microsphere size and its rates of formation and stabilization were related to the FND concentration, trapping laser power, and duration of irradiation. Using a fixed trapping laser power and duration, a higher FND concentration generally resulted in larger‐diameter aggregates in deionized water and shorter formation and stabilization times (Section S2.7 and Figure S7, Supporting Information). The dependence of the aggregate diameter on the trapping laser power and duration are shown in Figure 2d,e, respectively. The concentration of the FNDs in deionized water was fixed at 0.05 mg mL^−1^. Higher laser powers produced a stronger optical trapping force and thus larger FND aggregates with faster stabilization times (Figure 2d). Using a fixed trapping laser power (150 mW), larger‐diameter FND aggregates resulted from longer trapping durations (Figure 2e). For a given laser power, the diameter of the microsphere was limited by the attenuation of optical forces at the edge the trap, numerical investigations of which are presented later. As a result, prolonged irradiation did not increase the size of the aggregates indefinitely (insets I–III of Figure 2e). Larger‐diameter microspheres had a greater fluorescence intensity (Figure 2f; also see Video S1, Supporting Information), showing the effectiveness of using optically induced aggregation to enhance the FND fluorescence intensity.

By using an acousto‐optic deflector (AOD) in the SOTS, multiple FND microspheres could be simultaneously aggregated in a predesigned pattern using a customizable array of scanning optical traps. To demonstrate this, dispersed FNDs were aggregated in a customized array to form the word “NANO.” Then the array of microspheres was precisely transferred to a glass substrate (Figure 2g), where it was immobilized through van der Waals forces and became resistant to further movement (see Video S2, Supporting Information, for the patterning process). As intended, the fluorescence image of the patterned microspheres displays the word “NANO” (Figure 2h). In addition to FNDs, the aggregation method can be applied to nanoparticles created from other materials, such as metals or dielectrics (Figure S8, Supporting Information). Particularly, other temperature‐sensitive fluorescent nanoparticles, such as QDs and UCNPs, can be aggregated to form smaller spheres, enabling temperature detection with higher precision in terms of spatial location (Section S2.9 and Figures S9 and S10, Supporting Information).

### 2.3 Numerical Analysis of FND Microspheres

**Figure 3:**
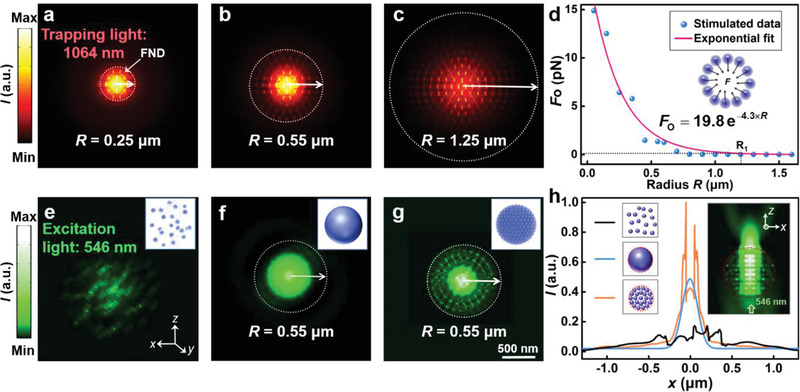
a–c) Energy intensity (*I*) in the *x*–*y* plane for the trapping light (wavelength: 1064 nm) applied to the aggregated FND microspheres with radii of 0.25, 0.55, and 1.25 µm. d) Optical forces on the FNDs at the outermost layer of the aggregated microspheres as a function of the radius *R*. The black dotted line indicates the critical radius (1.25 µm) of the aggregated microspheres. The inset shows the scheme of the optical forces exerted on the outermost layer of the microspheres. e–g) Energy intensity in the *x*–*y* plane for the excitation light (wavelength: 546 nm) illuminating the dispersed FNDs, a solid microsphere with a radius *R* of 0.55 µm, and an aggregated FND microsphere with the same *R*. The inset shows the three models for simulations. h) Energy intensity distributions along the *x* direction for the three models in (e–g). The inset shows the energy intensity of the excitation light in the *x*–*z* plane of the aggregated FND microsphere (*R* = 0.55 µm).

The aggregation mechanism of the FNDs was simulated using 3D finite element calculations (COMSOL Multiphysics 5.5, see the Experimental Section for further details) of the optical forces exerted on the FNDs (**Figure** **3**). The microsphere was modeled as a spherical aggregate of closely packed FNDs (diameter: 100 nm, refractive index: 2.4). The energy intensity (*I*) distributions in the *x*–*y* plane were simulated assuming a trapping laser wavelength of 1064 nm applied to microspheres with radii (*R*) of 0.25 (Figure 3a), 0.55 (Figure 3b), and 1.25 µm (Figure 3c). The resulting distributions indicate that the intensity at the outermost layer of microspheres with large radii is lower than those with small radii because the optical intensity of the trapping beam is radially attenuated. The optical force (***F*** o) exerted on an FND with surface area *S* can be expressed as^[^ ^49^ ^]^

(1) $$Fo=\oint_{S}\left(\langle T_{M}\rangle \cdot n\right)\mathrm{dS}$$

where ***n*** is the surface normal vector and 〈 ***T***_*M*_〉 is the time‐independent Maxwell stress tensor

(2) TM=12ReεEE*+μHH*−12εE2+μH2Q

and EE* and HH* are the outer products of the optical fields, *Q* is the unit dyadic, and *ε* and *μ* are the electric permittivity and magnetic permeability of the surroundings, respectively. We calculated the force on the FNDs at the outermost layer of the microspheres (Figure 3d), which varied exponentially with the radius (i.e., ***F*** o = ***A*** ⋅**e** *^−^* ***^B^*** *^R^*, where ***A*** and ***B*** are positive constants determined by the optical trap). The exponential relationship results from the radial attenuation of the optical intensity of the trapping beam. The experimental intensity distribution was also affected by the high refractive index of FNDs (*n* = 2.4) and the electromagnetic resonance between them, hence some points in Figure 3d deviate from the exponential curve. For a trapping laser power of 60 mW, the optical force was ≈0 for *R* > 1.25 µm; therefore, the upper limit on the FND microsphere diameter was ≈1.25 µm. The intensity distributions of the 546‐nm excitation light were also simulated to analyze the aggregation‐induced fluorescence enhancement. Distributions in the *x*–*y* plane were calculated for dispersed FNDs (Figure 3e), a solid microsphere with *R* = 0.55 µm (Figure 3f), and an aggregated FND microsphere with *R* = 0.55 µm (Figure 3g). The refractive index of the solid microsphere was assumed to be 2.4 (i.e., the same as that of the underlying FNDs). Line scans of the intensity along the *x* direction were performed for each of the above three cases (Figure 3h). Mie resonance occurred with the aggregated FND microsphere, which was attributed to the small size and high refractive index of the FND particles.^[^ ^50^, ^51^ ^]^ This resulted in an ≈7‐fold enhancement of the excitation light intensity in the microsphere compared with that in the dispersed FNDs (Figure S11, Supporting Information). Although the lensing effect of the solid microsphere can also enhance the excitation light intensity, the enhancement factor is only ≈2.8 because of the lack of electromagnetic resonance. More importantly, it is very difficult for such micrometer‐sized solid dielectric spheres to enter a living cell via endocytosis. Thus, to obtain a given fluorescence emission intensity for the detection of multiple regions, a lower excitation power could be applied to the aggregated microspheres compared with dispersed single nanoprobes,^[^ ^42^, ^43^, ^44^, ^45^, ^46^ ^]^ thereby reducing photodamage to the cells (Section S3.2 and Figure S12, Supporting Information).

### 2.4 Intracellular Aggregation, Patterning, and Thermal Probing

**Figure 4:**
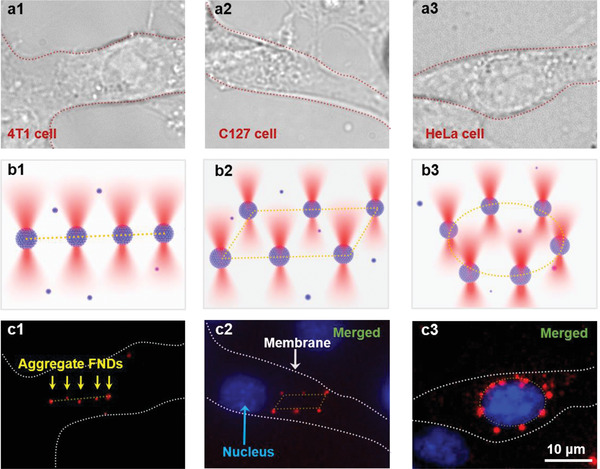
a) Confocal microscope images of 4T1 (a1), C127 (a2), and HeLa cells (a3). The red dashed lines represent the outlines of the cancer cells. b) Customized scanning optical trap arrays in the patterns of a chain (b1), a parallelogram (b2), and an ellipse (b3). c) Fluorescence images of the aggregated FND microspheres in customized trap arrays in the cells. The aggregated FND microspheres were patterned into a chain in the 4T1 cell (c1), a parallelogram near the nucleus of the C127 (c2), and an ellipse encircled the HeLa nucleus (c3). Red: the aggregated FND microspheres excited by 546‐nm laser. Blue: nuclei stained with Hoechst 33342 live nuclei stain (excitation: 350 nm, emission: 461 nm). White dashed line: the membranes of the cancer cells.

The FND microspheres could be formed and arranged intracellularly using the SOTS. To demonstrate this, we performed the intracellular aggregation and patterning of customized FND microsphere arrays in 4T1 (**Figure** **4**a1) and C127 (Figure 4a2) breast cancer cells, and in HeLa cervical cancer cells (Figure 4a3). The FNDs readily permeate adherent cells in culture^[^ ^52^ ^]^ and have the least cytotoxic effects of all carbon materials.^[^ ^16^ ^]^ After addition and incubation of the FND solution (particle diameter: 100 ± 30 nm, concentration: 30 µg mL^−1^) for 4 h in culture (see the Experimental Section for further details), customized arrays of scanning optical traps (power: 150 mW) were applied to 4T1, C127, and HeLa cells to aggregate the cytoplasmic dispersion of FNDs into the shape of a chain (Figure 4b1), parallelogram (Figure 4b2), and ellipse (Figure 4b3), respectively. The customized arrays trapped the FNDs dispersed in the cytoplasm to form the microspheres in the desired patterns. Compared with an isolated solution, formation of stable FND aggregates in the complex intracellular environment required a higher trapping laser power (150 mW) and a longer duration of irradiation (≈180 s). Despite this, the intracellular FND aggregates remained stable after the laser was turned off (Figure S13, Supporting Information). After aggregation and patterning, the FND microspheres were excited with 546‐nm light and fluorescence images displaying the corresponding intracellular patterns were obtained (Figure 4c; see Video S3, Supporting Information, for the intracellular aggregation and patterning processes). Cell nuclei were labeled using Hoechst 33342 fluorescent dye (excitation: 350 nm, emission: 461 nm) and no fluorescence intensity changes or fragmentation of nuclei were observed after aggregation and patterning, indicating that the cells remained viable (see the Experimental Section for further details).^[^ ^53^ ^]^

The parallelogram‐shaped array of the FND microspheres was arranged adjacent to the C127 cell nucleus (Figure 4c2), whereas the elliptical array encircled the HeLa nucleus (Figure 4c3). The size of the FND microspheres near the nucleus was larger than that in the cytoplasm because the uptake of FNDs was higher around the nucleus of the HeLa cell. Differences in FND endocytosis among the cell types also affected aggregate formation (Section S4.2 and Figure S14a,b, Supporting Information). Moreover, different cell types and compartments have unique microenvironments that affect the final size, stabilization time, and fluorescence intensity of the FND aggregates (Section S4.2 and Figure S14c–e, Supporting Information). The aggregates could be directionally moved in a small area of the cell by the SOTS to achieve the thermal probing in desired positions or organelles (Figure S15, Supporting Information). Moreover, to investigate the possible cytotoxic effects on the cells caused by the trapping laser or the FND aggregates, experiments were carried out to indicate the cell viability after trapping the FNDs by the optical tweezers in living cells for different trapping powers, irradiation durations and amounts of FND aggregates, showing that the endocytosis of the FNDs has little effect on the cell survival because of the good biocompatibility (see Section S4.4 and Figure S16, Supporting Information, for details).

**Figure 5:**
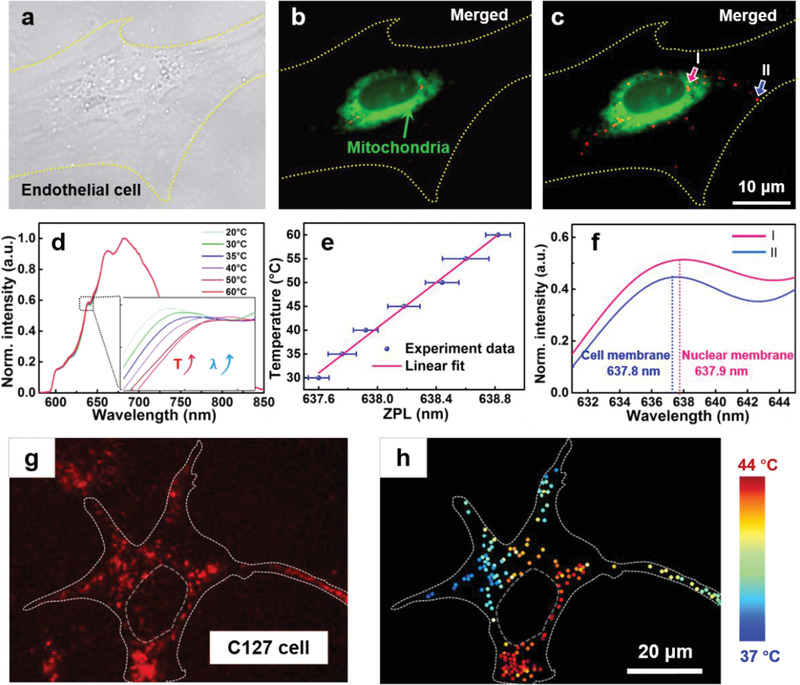
a) Confocal microscope image of a human brain microvascular endothelial cell (HMEC‐1). b) Fluorescence image of the cell with mitochondria stained by Mito‐Tracker Green FM (excitation: 490 nm, emission: 516 nm). c) Fluorescence image of the FNDs aggregated and patterned at different locations in the HMEC‐1. Blue and pink arrows pointed to the microspheres near the nuclear membrane and cell membrane, respectively. d) Fluorescence spectra of the FNDs illuminated by the 546‐nm laser in solution at different temperatures. The inset is an enlarged view of the temperature‐induced shift of the ZPLs of the spectra obtained at 20–60 °C. e) Calibration of the ZPL at each temperature from 20 to 60 °C. Error bars represent the mean standard deviation of the multi‐particle ZPL wavelength. f) Fluorescence spectra of the aggregated FND microspheres close to the nuclear membrane and cell membrane of the HMEC‐1, indicated by I and II in (c), respectively. g) Fluorescence image of FND aggregates at different locations in a living C127 cell. h) Temperature mapping of the cell with the FND aggregates.

Intracellular thermal probing was next demonstrated by measuring the fluorescence signal from aggregated FND microspheres at different locations within a human brain microvascular endothelial cell (HMEC‐1) (**Figure** **5**). After 24 h in culture (Figure 5a), the mitochondria were stained with Mito‐Tracker Green FM (Figure 5b) to help visualize the approximate temperature distribution within the cell (see the Experimental Section for further details). The FNDs were then endocytosed and a trapping laser with a power of 150 mW was applied at different intracellular locations identified in the bright‐field image. With such a laser power, the precisions of placing the microsphere in the *x*, *y*, and *z* directions were 27, 28, and 56 nm, respectively (see Section S4.5 and Figure S17, Supporting Information, for the detailed description of the precision). After 3 min of laser irradiation, the FNDs were intracellularly aggregated at different locations of the endothelial cell (Figure 5c). Before temperature probing, the FNDs were excited using 546‐nm light. The NV^−^ center causes a broad emission that peaks at 685 nm and changes very little between 20 and 60 °C. However, due to the electronic transition in the NV^−^ centers (^3^A_2_→^3^E), the ZPL at 637 nm exhibits a redshift as the solution temperature increases (Figure 5d). By measuring the ZPLs of four aggregated FND microspheres at each temperature between 20 and 60 °C (Figure 5e) (see Section S4.6 and Figure S18, Supporting Information, for an example of the temperature analysis with four aggregates), the thermal shift (sensitivity) was found to be 25 °C nm^−1^. Using a high‐resolution spectrometer (ANDOR, Shamrock 750, resolution: 0.02–0.04 nm, accuracy: 0.03 nm) assisted by an optical‐microscope‐based diaphragm (CRAIC, 20/30 PV), the fluorescence spectra of the aggregated FND microspheres could therefore be used to measure a minimum temperature difference (Δ*T*_min_) of 0.5 °C at the desired locations. Fluorescence spectra were then obtained for the aggregated FND microspheres close to the nuclear membrane (Figure 5c, location I) and outer membrane (Figure 5c, location II) of the endothelial cell (Figure 5f). The distance between the aggregated FND microsphere and the nuclear membrane was ≈0.20 µm, and the corresponding distance from the cell membrane was ≈0.27 µm. The measured ZPL shifted to 637.8 and 637.9 nm at locations I and II, respectively, indicating that the temperature at the nucleus was 2.5 °C higher than that at the membrane. This is because most of the energy‐generating mitochondria stained were gathered around the nucleus.^[^ ^54^ ^]^ Using the same approach, temperature mapping in a living C127 cell was also performed (Figure 5g,h). The mapping indicates the general distribution of the mitochondria within the C127 cell, providing information about its physiological activity. The above results demonstrate the effectiveness of using aggregated FNDs to perform site‐specific intracellular thermal probing.

## 3 Conclusion

We described an optical method for intracellular FND aggregation and demonstrated that the resulting FND microspheres are thermal probes capable of spatially resolving intracellular temperature differences. The endocytosed FNDs were aggregated and positioned with high precision and flexibility, and the excitation light intensity was markedly enhanced by the FND aggregation and electromagnetic resonance within the microspheres. Using the enhanced fluorescence signals from the FNDs, spectra were obtained to determine the temperature at different locations in living cells. The methods can be broadly applied to intracellular analyses and the construction of intracellular nanophotonic devices.

## 4 Experimental Section

### FND Characterization

The FNDs (catalogue number: NDNV100nmHi, customization: undecorated, Adamas Nanotechnologies, USA) were made from diamonds synthesized under high pressure and temperature. Because FNDs were produced by crushing micrometer‐sized diamonds, they had an irregular shape. The equivalent diameter of FNDs was measured using a SEM (Apreo, Thermo Fisher Scientific, USA) collecting secondary electrons in the immersion mode at 2.00 kV, 25 pA, and a working distance of 2.0 mm. A T3 detector was positioned close to the sample to obtain images of the FNDs. The morphology and size of ≈100 FNDs with sheet‐like structures were then determined from the SEM images, yielding a statistical equivalent diameter of 100 ± 30 nm. The FNDs were soluble in deionized water. Although the surface of an FND with diameter greater than 50 nm is considered to be chemically inert, it usually contains oxygen‐containing functional groups resulting from the use of strong oxidants during the purification process. In the Fourier‐transform infrared (FTIR) spectra measured by an FTIR spectrometer (Spectrum Two, Perkin‐Elmer, USA) (Figure S1a, Supporting Information), absorption peaks were observed at 1639 cm^−1^ (typical of C═C group stretching vibrations) and 3328 cm^−1^ (corresponding to stretching vibrations of hydroxyl groups).^[^ ^55^ ^]^ The zeta potential measured by a particle size and potential analyzer (NanoBrook Omni, Bookhaven, USA) was −8 mV, indicating that the surface hydroxyl groups were negatively charged (Figure S1b, Supporting Information).^[^ ^56^ ^]^ Because the color center is embedded in the FND matrix, its fluorescence characteristics are not affected by surface modifications. In the Raman spectrum of the FND aggregate measured by a Raman microscope system (XploRA PLUS, Horiba Jobin Yvon, Japan) (Figure S1c, Supporting Information), a sharp sp^3^ peak at 1332 cm^−1^ was observed, indicating the presence of a significant diamond crystal core.^[^ ^57^ ^]^ In addition, the absorption spectrum measured by a UV–vis–NIR spectrometer (Lambda750, Perkin‐Elmer, USA) and the emission spectrum measured by a high‐resolution micro‐spectrometer (HR2000, Ocean Optics, USA) of the FNDs (Figure S1d, Supporting Information) revealed absorption and emission peaks at 550 and 685 nm, respectively.

The number of the NV^−^ centers was obtained using electron paramagnetic resonance. Experiments were performed at X‐band (9.87 GHz) and Q‐band (34 GHz) using a Bruker ELEXSYS E500 spectrometer. The samples were weighed and inserted in a quartz tube, then the spectra were recorded under non‐saturating conditions using a Bruker Super High Q (SHQE) cavity. A spectrum of powdered 2,2‐diphenyl‐1‐picrylhydrazyl was recorded using the same temperature and acquisition parameters and used as a concentration standard to estimate the number of spins in the samples. Each NV^−^ center is oriented such that its axis is parallel to one of the four 〈111〉 crystallographic directions. In the EPR measurements, the magnetic field was aligned along the 〈111〉 direction. In this configuration, the observed EPR spectrum consists of two pairs of lines displaced symmetrically with a Lande factor of 2.0028. These measurements determined that the NV^−^ concentration was ≈300 centers per 100‐nm particle.

### Experimental Setup

The experiment setup was constructed around a SOTS (Tweez250si, Aresis Co., Ltd., Slovenia), and a computer‐controlled AOD system was used to adjust the position and strength the optical traps (trap position resolution: 0.01 nm). A continuous‐wave, 1064‐nm laser beam (maximum input power: 5 W) emitted by a Nd:YAG laser was passed through the AOD and a beam expander, reflected upward by a dichroic mirror, then transmitted into the microscope. The expanded beam and an RGB LED source with a center wavelength of 546 nm were coupled into the optical pathway of the microscope using the dichroic mirror, then tightly focused into the sample chamber using a 60× water‐immersion inverted objective (numerical aperture: 1.0). The experimental process could be captured by a high‐speed CCD camera and monitored on a computer screen in real time.

To investigate the aggregation process, an optical trap created by the SOTS was applied to the solution of FNDs (solvent: deionized water, concentration: 0.05 mg mL^−1^) dispersed in a 100‐μm‐thick sample chamber. Because of the continuous action of the optical force, the FNDs were trapped near the center of the beam and an aggregated FND microsphere with a diameter of 2 µm was formed within 12 s using a trapping power of 150 mW. Finally, a stable aggregated FND microsphere was formed by the electrostatic attraction between the FNDs after the laser was turned off.

### Cell Culture

The 4T1 (mouse breast tumor), C127 (mouse breast tumor), and HeLa (cervical cancer) cell lines (Science Cell Research Laboratories, USA) were cultured in Roswell Park Memorial Institute (RPMI) 1640 medium, Dulbecco's modified Eagle medium (DMEM), and minimum essential medium (MEM), respectively. The media were supplemented with 10% fetal bovine serum (FBS) and 1% penicillin/streptomycin (P/S) solution. The cells were cultured in a humidified incubator at 37 °C with 5% CO_2_ to achieve ≈70% confluency after 24 h. The HMEC‐1 cells (Science Cell Research Laboratories, USA) were cultured in endothelial cell medium supplemented with 5% FBS, 1% P/S, and endothelial cell growth supplement in a humidified incubator at 37 °C with 5% CO_2_ for 24 h.

### Internalization of FNDs, Cell Viability Assays, and Staining of Mitochondria

The 4T1, C127, HeLa, and HMEC‐1 cells were seeded in 20‐mm‐diameter confocal dishes and cultured for 24 h to reach ≈70% confluency. The 100‐nm FNDs were dispersed in fresh medium at a concentration of ≈30 µg mL^−1^ and the solution was UV‐sterilized for 30 min. The cell monolayer was washed three times with PBS, then treated with the FND solution in a humidified incubator at 37 °C and 5% CO_2_ for 4 h. Finally, the cells were rinsed with complete medium to remove the extracellular FNDs.

The tumor cells were stained with Hoechst 33342 dye (Beyotime Institute of Biotechnology, Shanghai) for 30 min at a final concentration of 2 mg mL^−1^. After labeling, the cells were washed with PBS to remove the excess dye, and the cell viability was evaluated using a fluorescence microscope with excitation and emission wavelengths of 350 and 461 nm, respectively. In healthy cells, the nuclei are approximately spherical and the DNA is evenly distributed. Binding of the fluorescent dye to DNA can be used to observe the nuclear morphology after aggregation and patterning of intracellular FNDs. Only a small amount of dye can enter the normal cell membrane and stain DNA, yielding a uniform, low‐intensity blue fluorescence. However, the aggregation of DNA during apoptosis also causes the dye to aggregate, resulting in a fluorescence intensity much higher than that observed in normal cells.

The mitochondria of HMEC‐1 cells were stained with Mito‐Tracker Green FM (100 × 10^−9^ m, Beyotime Institute of Biotechnology, Shanghai) for 30 min, then washed with medium to remove the free dye. After incubation, the cultures were observed using a fluorescence microscope with excitation and emission wavelengths of 490 and 516 nm, respectively.

### Simulations

In the 3D simulations with COMSOL Multiphysics 5.5, a radio frequency module (electromagnetic wave, frequency domain) and a perfectly matched layer boundary condition were applied. The trapping laser was set as an unpolarized Gaussian beam at a wavelength of 1064 nm (power: 60 mW), and the FNDs were set as spheres with a diameter of 100 nm. The refractive indices of water and the FNDs were set as 1.33 and 2.40, respectively, and the corresponding mesh sizes were 80 and 50 nm. To analyze the excitation light intensity distributions of the dispersed FNDs, solid microsphere and aggregated FND microsphere, the excitation light was set as a plane wave at a wavelength of 546 nm, and the mesh sizes of water and FNDs were set as 60 and 40 nm, respectively.

### Statistical Analysis

All data statistical analyses were presented as mean ± standard deviations of at least three independent experiments. Analysis was performed using Origin software (version: 2018b, OriginLab Inc., USA). The number of samples for each analysis was introduced in each figure legend.

## Conflict of Interest

The authors declare no conflict of interest.

## Supporting information

Supporting Information

Supplemental Video 1

Supplemental Video 2

Supplemental Video 3

## Data Availability

Research data are not shared.
