# Supplementary material for: Intracellular Thermal Probing Using Aggregated Fluorescent Nanodiamonds
Source: Adv Sci (Weinh). 2021 Nov 23;9(3):2103354. doi: 10.1002/advs.202103354 (PMC8787390; doi:10.1002/advs.202103354)
Supplement: Supplementary file 1 — Supporting Information [file ADVS-9-2103354-s002.pdf]

ADVANCED SCIENCE
Open Access

Supporting Information

for *Adv. Sci.*, DOI: 10.1002/advs.202103354

Intracellular Thermal Probing using Aggregated Fluorescent Nanodiamonds

Tianli Wu[1], Xixi Chen[1], Zhiyong Gong[1], Jiahao Yan[1], Jinghui Guo[2], Yao Zhang[1], Yuchao Li[1*] and Baojun Li[1*]*

Supporting Information for

Intracellular Thermal Probing using Aggregated Fluorescent Nanodiamonds

Tianli Wu[1], Xixi Chen[1], Zhiyong Gong[1], Jiahao Yan[1], Jinghui Guo[2], Yao Zhang[1*],

Yuchao Li[1*] and Baojun Li[1*]

[1]*Institute of Nanophotonics, Jinan University, Guangzhou 511443, China*

[2]*Department of Physiology, School of Medicine, Jinan University, Guangzhou 510632, China*

[*]*email: zhyao5@jnu.edu.cn (Y.Z.), liyuchao@jnu.edu.cn (Y.L.), or baojunli@jnu.edu.cn (B.L.)*

S1. Temperature-sensitive nanomaterials

Parameter	Size	Excitation/ Emission	Material	Temperature precision	Fluorescence quantum yield	Photobleaching
QDs	~ 5 nm	360/485 nm	Semiconductor	0.16 °C[S1]	> 90 %	Yes
UCNPs	~ 35 nm	975/545 nm	Rare earth	0.20 °C[S2]	~ 5.0 %	None
FNDs	~ 100 nm	546/685 nm	Carbon	0.10 °C[S3]	> 90 %	None

Table S1. Comparison of the fluorescent nanoparticles for temperature detection.

S2. Formation of the aggregates

S2.1. FND Characterization

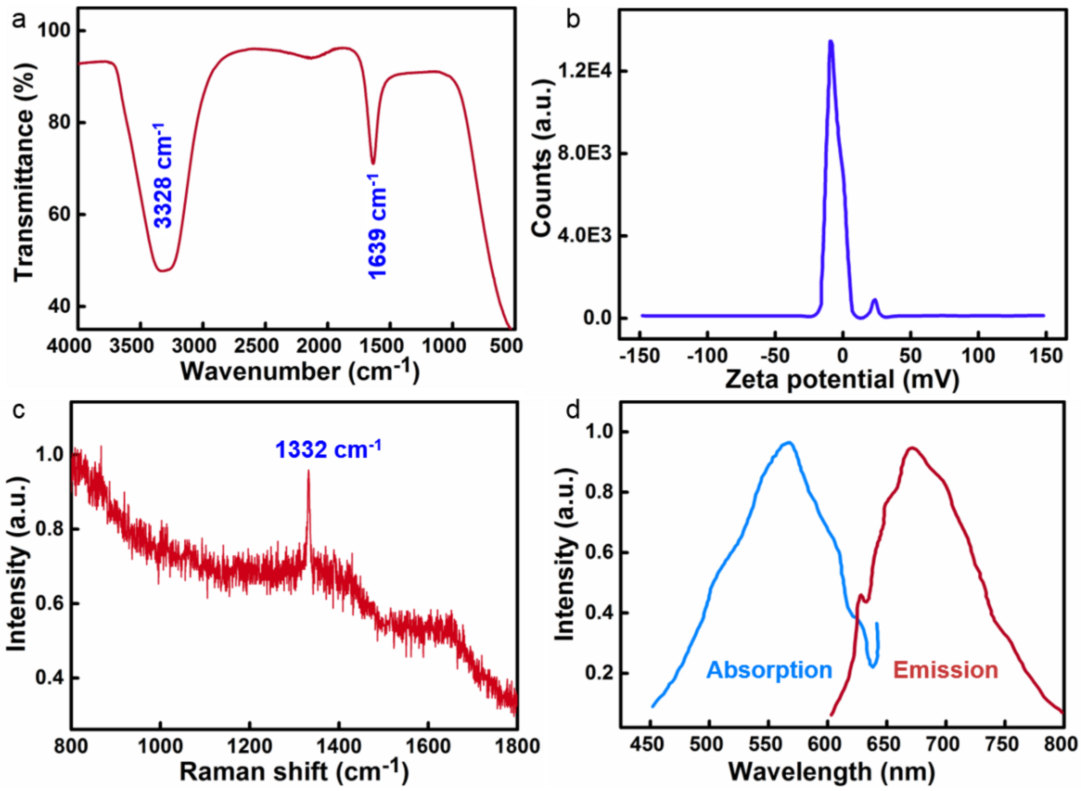

Figure S1. a) Fourier-transform infrared spectra (FTIR) of the FNDs with diameter of 100 nm. b) Zeta potential of the FNDs in deionized water. c) Raman spectrum of the FNDs acquired with an excitation laser at 785 nm. d) Absorption and emission spectra of the FNDs.

S2.2. Optical trapping stiffness of FND aggregates

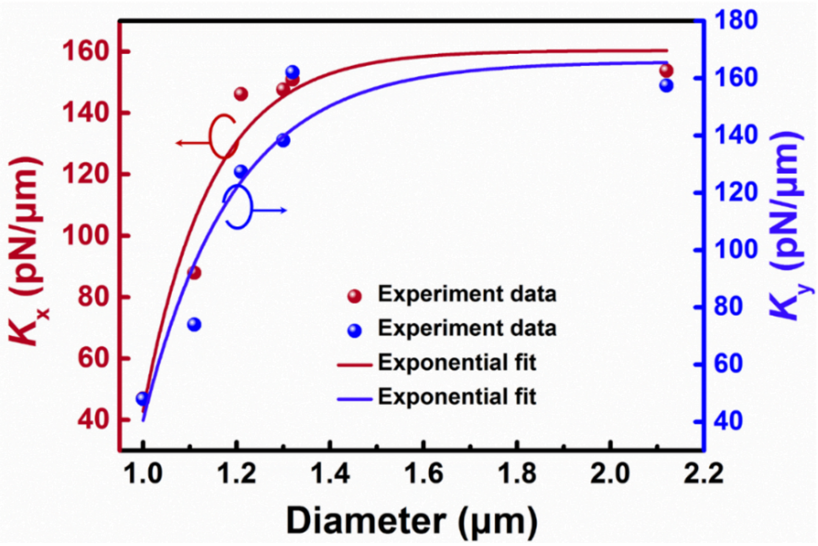

Figure S2. Optical trapping stiffness of the FNDs in aggregation (in *x* and *y* directions).

S2.3. Stability of aggregated FND microspheres

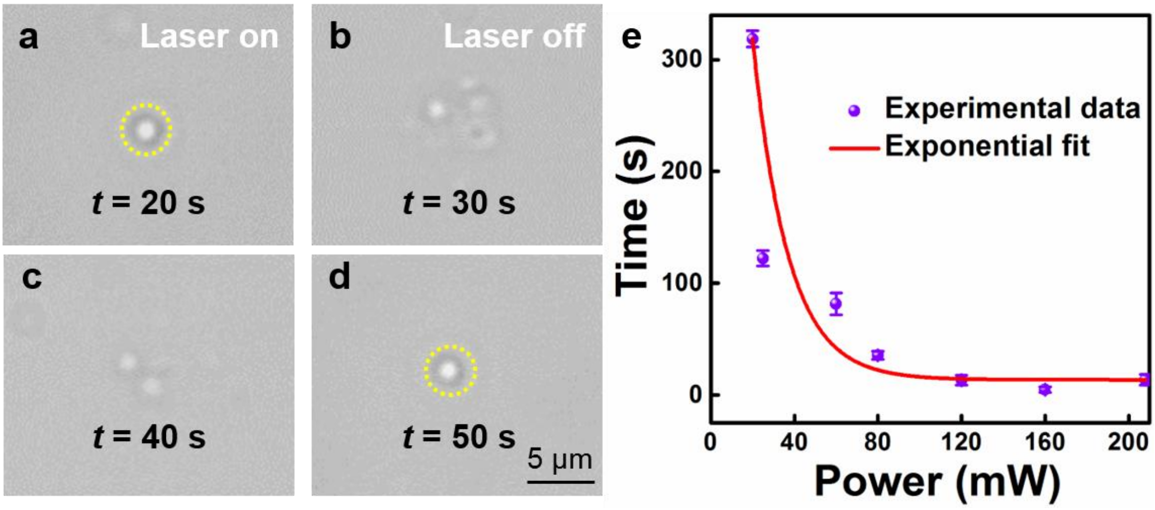

Figure S3. a) An FND microsphere was gradually formed and the diameter reached 1.5 μm after switching on the trapping laser at 50 mW for 20 s. b, c) The FND microsphere was gradually scattered after switching off the laser for 30 (b) and 40 s (c). d) The FND microsphere became stabilized after aggregation of 50 s and maintained the spherical structure even if the trapping laser was removed. e) Relationship of the optical power and stabilization time of the aggregated microspheres.

S2.4. Electrostatic adsorption in aggregated FNDs

As presented in Figure S1, there exist negatively charged −OH groups (−8 mV) on the surface of the FNDs in deionized water, which results in the electrostatic repulsion between the FNDs. Theoretically, when the electrostatic forces between the particles is repulsive, an increase in ionic strength will rise the absorption capacity.[S4] The experiments on the forming and stabilization time of the aggregated FND microspheres in NaCl solution at different concentrations were carried out, as shown in Figure S4. The trapping laser power was set as 100 mW, and the FND concentration is 0.05 mg/ml. As a control group, the formation and stabilization times of the FND aggregates were 20 and 38 s with a diameter of 2.0 μm in deionized water, respectively. With the concentration of the NaCl solution increased to 0.01 (inset I of Figure S4) and 0.02 mol/L (inset II of Figure S4), the formation time was shortened to 4.2 and 4 s while the stabilization time was shortened to 12 and 5 s, respectively. Note that

as the concentration of the NaCl was further increased to 0.03 mol/L (inset III of Figure S4), the strong electrostatic adsorption between the FNDs caused an aggregation in large numbers and the spherical shape of the aggregate was no longer maintained.

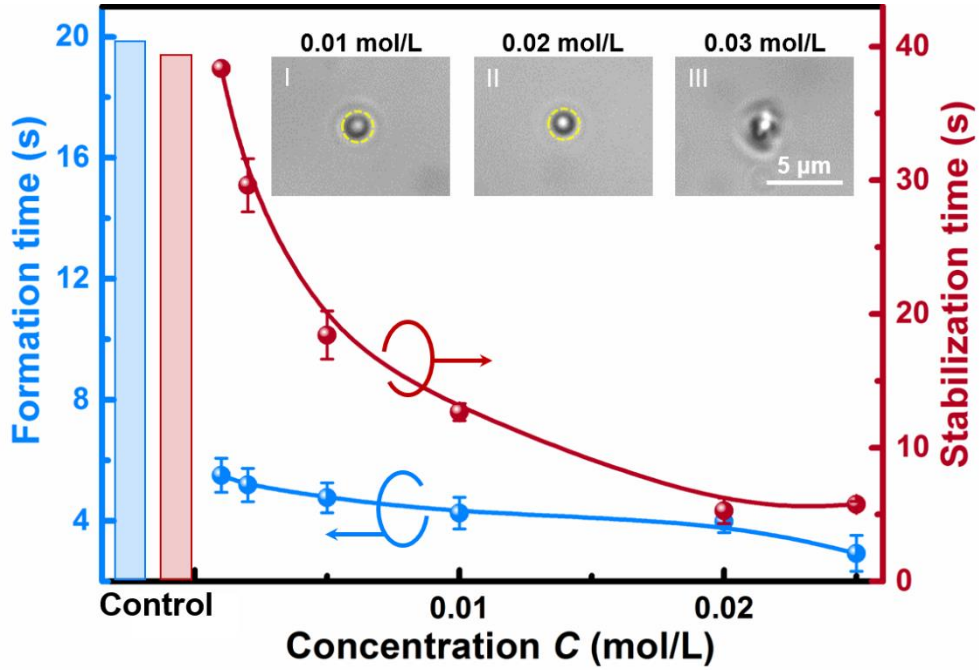

Figure S4. Formation and stabilization times of the aggregated FND microspheres in NaCl solution at different concentrations. Data obtained in deionized water were presented as a control group. Insets (I–III): Confocal microscope images of the aggregated FNDs in solution at concentrations of 0.01 (I), 0.02 (II), and 0.03 mol/L (III). The aggregate in (III) no longer maintained a spherical shape.

S2.5. Roundness of aggregated FND microspheres

The roundness of the aggregated FND microspheres is evaluated by estimating the roughness acquired from the SEM images (Figure S5). Here the roughness is defined as the average difference between the diameters of the FND microspheres and the diameter of an absolute sphere. The results show that the roundness gets smaller with the increase of the diameter of the aggregated FND microspheres (Figure S5e).

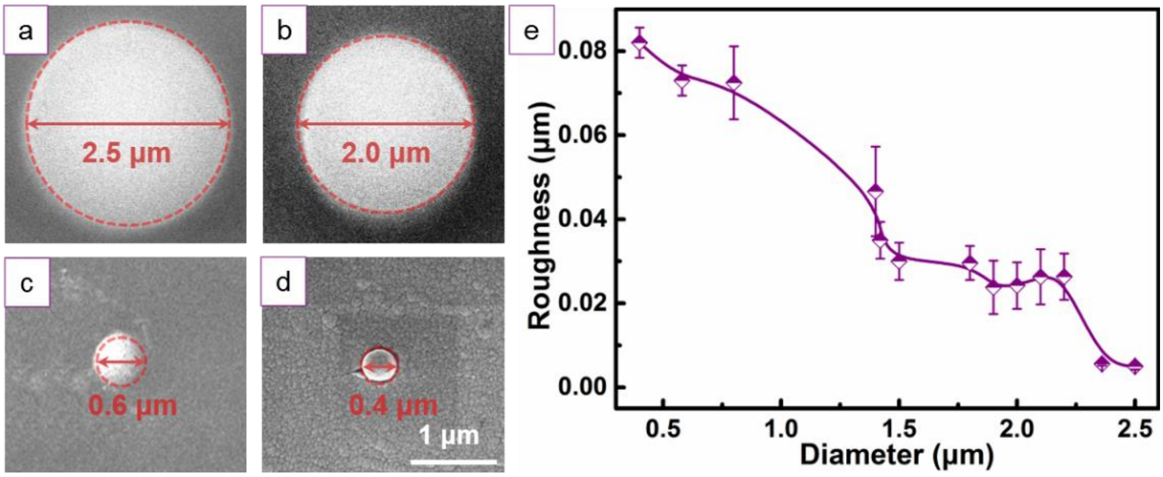

Figure S5. a-d) The SEM images of aggregated FND microspheres with diameter of 2.5 (a), 2.0 (b), 0.6 (c) and 0.4 µm (d), respectively. e) Statistics of roughness and diameter of the aggregated FND microspheres.

S2.6. Minimum size of stable FND aggregates

The minimum size of the stable aggregates was 0.4 µm, which was formed by applying a trapping laser power of 50 mW within irradiation duration of 5 s, as shown in Figure S6. Theoretically, smaller aggregates can be obtained with lower trapping power or shorter irradiation time. However, the roughness of the aggregates, as to be discussed previously (Figure S5), will be higher than 0.08 µm, which cannot be considered as a spherical structure. Therefore, the smallest size of aggregate FND microsphere was 0.4 µm that contained ~800 individual FND particles to ensure a satisfactory roundness.

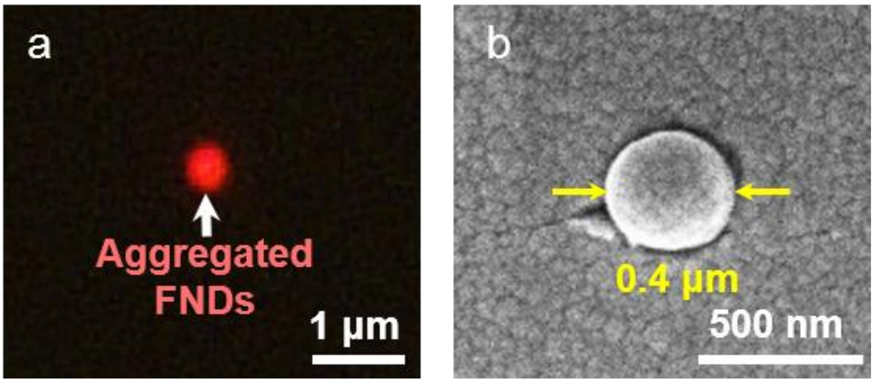

Figure S6. a) Fluorescence image of the FND aggregated microsphere excited by 546 nm. b) SEM image of the minimum FND aggregated microsphere.

S2.7. Aggregation process at different FND concentrations

The FND concentration affects the aggregate diameter and the formation and stabilization time, as shown in Figure S7. At a fixed power of 150 mW and an irradiation time of 5 s, the diameter of the FND aggregates gradually increases with the concentration (Figure S7a), while the formation and stabilization time decreases (Figure S7b).

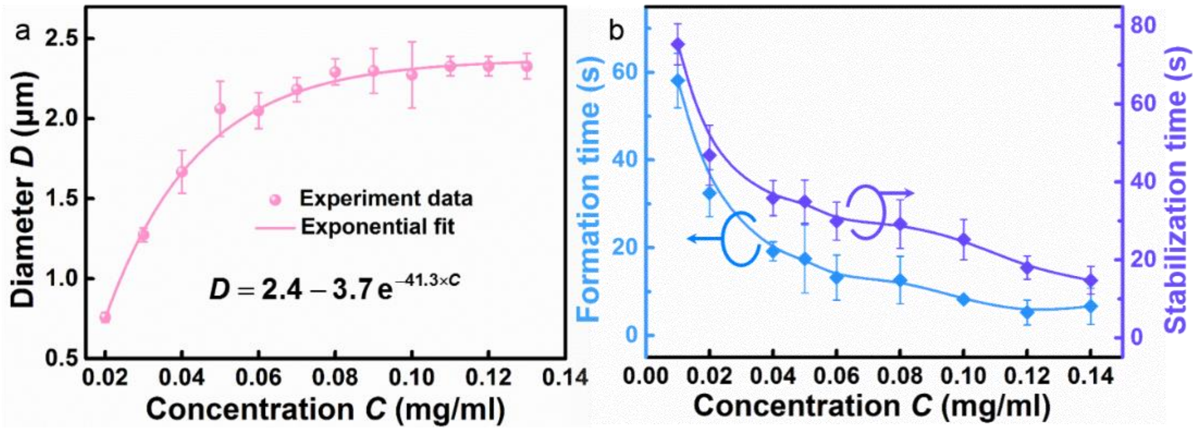

Figure S7. a) Aggregated FND microsphere diameters as a function of the concentration. b) Statistics of the formation and stabilization time of the aggregated FND microspheres at different concentrations.

S2.8. Optical aggregation of nanoparticles of other materials

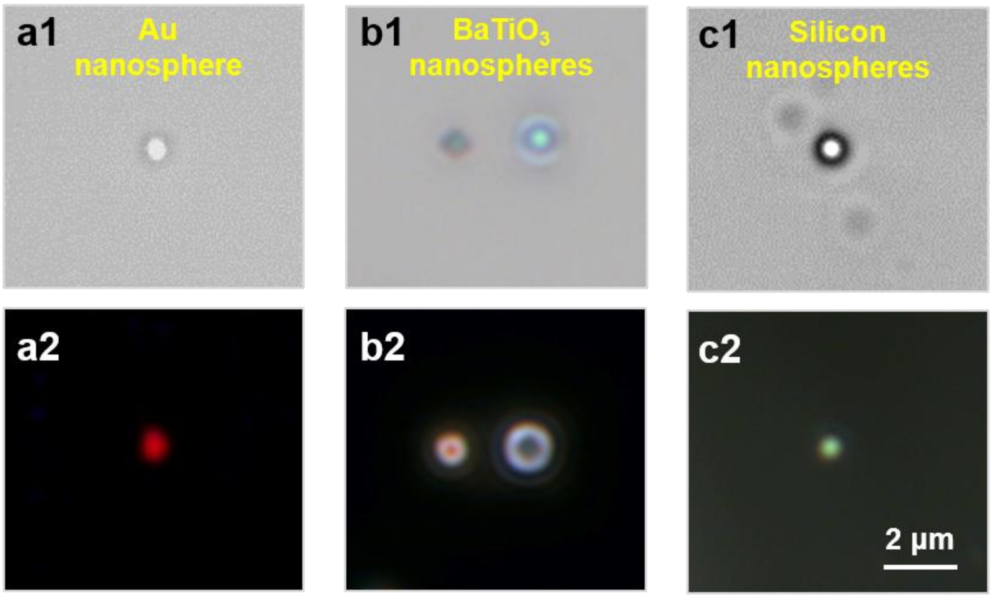

Figure S8. Bright-field microscope and fluorescence images of aggregated Au (a1,2), $BaTio_3$ (b1,2) and Si nanospheres (c1,2) with diameters of 60, 100, and 80-200 nm, respectively.

S2.9. Aggregation of other temperature-sensitive nanoparticles

Although the size of aggregates can be controlled by the concentration, trapping laser power and irradiation duration, it is strongly related to the size of the individual nanoparticles that form the aggregates. As shown in Figure S9, dispersed quantum dots (QDs, diameter: 5 nm) and upconverting nanoparticles (UCNPs, diameter: 35 nm) (Figure S9a) were aggregated into spheres by the trapping laser, emitting blue and green light under the excitation of 380 and 980 nm lasers, respectively (Figure S9b). The SEM images in Figure S9c show that the QD and UCNP aggregates had regular spherical shapes and their diameters were 70 and 360 nm, respectively. These fluorescent aggregates are also temperature-sensitive and can be used for thermometry. For example, by measuring the fluorescence spectra of an aggregated UCNP sphere in solutions at different temperatures (Figure S10a), the fluorescence intensity radio (*FIR*) at 525 and 548 nm was obtained (Figure S10b), which can be used for the temperature detection of desired locations. Note that although the aggregates of other temperature-sensitive fluorescent nanoparticles are also effective in temperature detection, FND aggregates were chosen in this work for intracellular probing and the reason was previously discussed (Table S1).

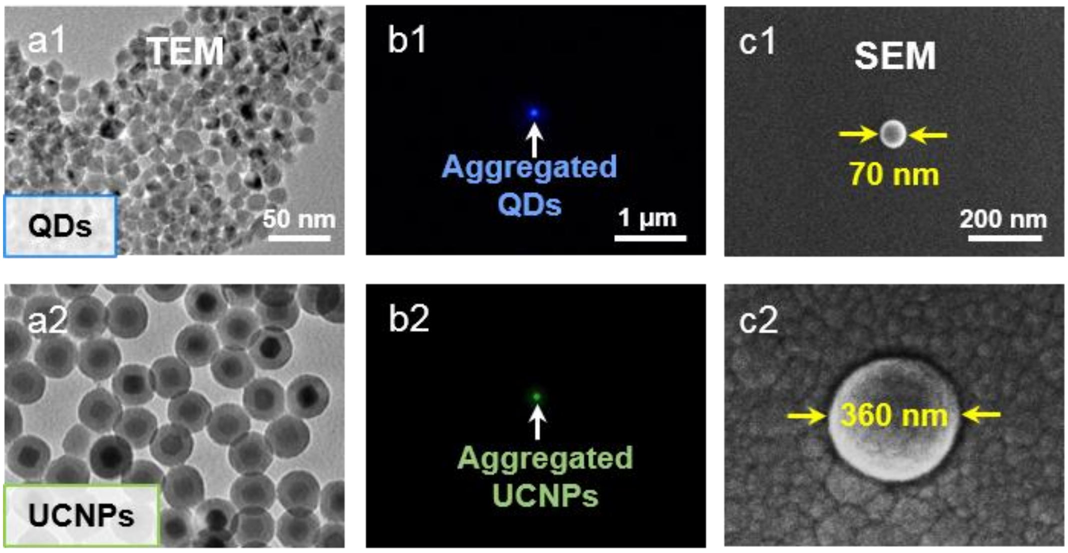

Figure S9. a) Transmission electron microscope images of QDs (a1) and UCNPs (a2). b) Fluorescence images of the aggregated QD (b1) and UCNP (b2) spheres excited by 380 and 980 nm, respectively. c) SEM images of the QD (c1) and UCNP (c2) aggregates.

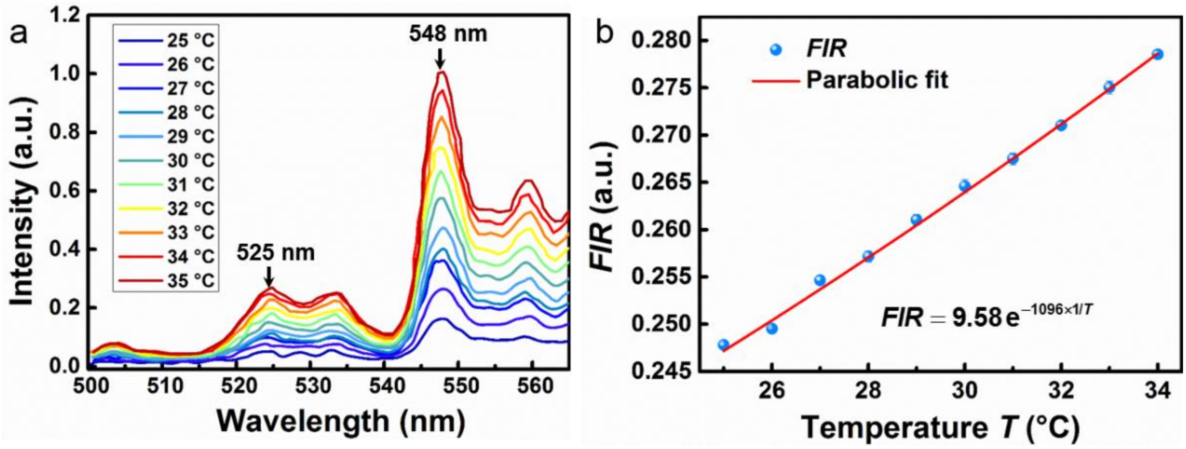

Figure S10. a) Upconversion emission spectra showing the evolution of fluorescence intensities at different temperatures. b) Fluorescence intensity ratio at 525 and 548 nm as a function of temperature.

S3. Numarical analysis and comparative experiments

S3.1. Simulations and calculations

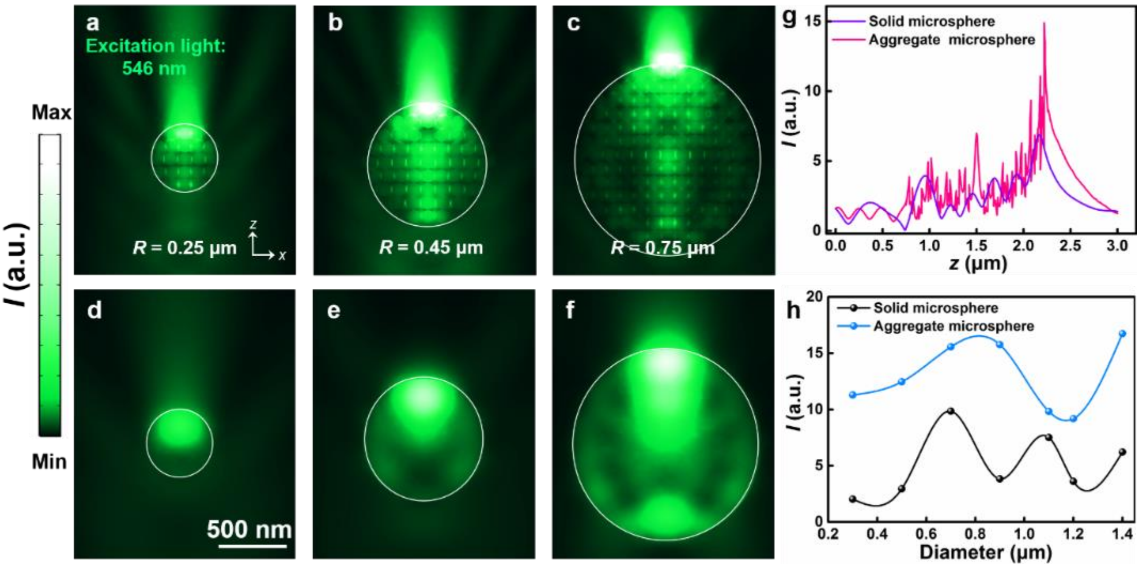

Figure S11. a-c) Energy intensity (*I*) of the excitation light (546 nm) on aggregated microspheres with radii of 0.25 (a), 0.45 (b) and 0.75 μm (c) in the *x*-*z* plane. d-f) Energy intensity of the excitation light (546 nm) on solid microspheres with the same radii of (a-c), in the *x*-*z* plane. g) Energy intensity distributions along the *z* direction of the microspheres with the radius of 0.75 μm. The pink and purple lines indicate the aggregated FND microsphere and a single solid microsphere, respectively. Fringes exist in the pink line due to the interference between the incident and the emitted light by the FNDs. h) Maximum energy intensities of the aggregated FND microsphere and the single solid microsphere as functions

of the diameter.

S3.2. Comparision of aggregated and dispersed nanoparticles

For the dispersed single probes, such as FNDs (100 nm), UCNPs (35 nm) and QDs (5 nm), the fluorescence intensity (I_0) is usually weak so that the fluorescence imaging and detection are relatively difficult (Figure S12a). By forming the dispersed probes into the aggregates, the fluorescence intensity (I_1) becomes much stronger at the same excitation power (Figure S12b). Statistically, the enhancement factor f of the fluorescence intensity, defined as $f = I_1/I_0$, is at least 4.3 (Figure S12c). This means although the single probes dispersed in the cells can be

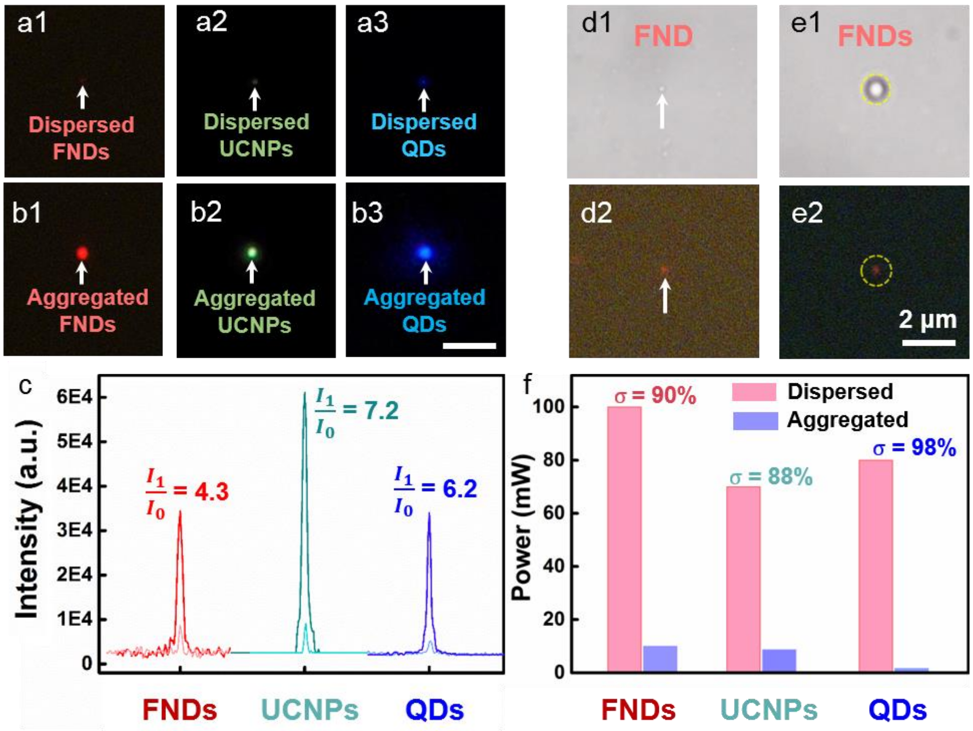

Figure S12. a) Fluorescence images of dispersed FNDs (a1), UCNPs (a2), and QDs (a3) under the excitation of 546, 980 and 380 nm, respectively. b) Fluorescence images of the aggregated FND (b1), UCNP (b2), and QD (b3) spheres under the same excitation wavelength and power with those in (a), scale bar: 2.0 μm. c) Normalized fluorescence intensity of (a, b). I_0 and I_1 are the fluorescence intensities of the dispersed particles and the aggregates with the same excitation power, respectively. d) Bright-field and fluorescence image of a dispersed FND. e) Bright-field and fluorescence image of an aggregated FND microsphere (diameter: 1.0 μm). The fluorescent intensity in (e2) is the same with that in (d2). f) Excitation powers for the same emission intensity in different dispersed and aggregated fluorescent nanoparticles.

The power reduction σ is defined as $\sigma = (P_0 - P_1)/P_0$, where P_0 is the excitation power for the dispersed nanoparticles and P_1 is the excitation power for the aggregates.

targeted to specific locations, they require a relatively high excitation power to detect multiple regions, which may cause certain damage to the cell. With the method of aggregating the FNDs, the excitation power can be reduced by up to 90% to obtain the same fluorescence intensity (Figure S12d-f).

S4. Intracellular experiments

S4.1. Stability of aggregates in cells

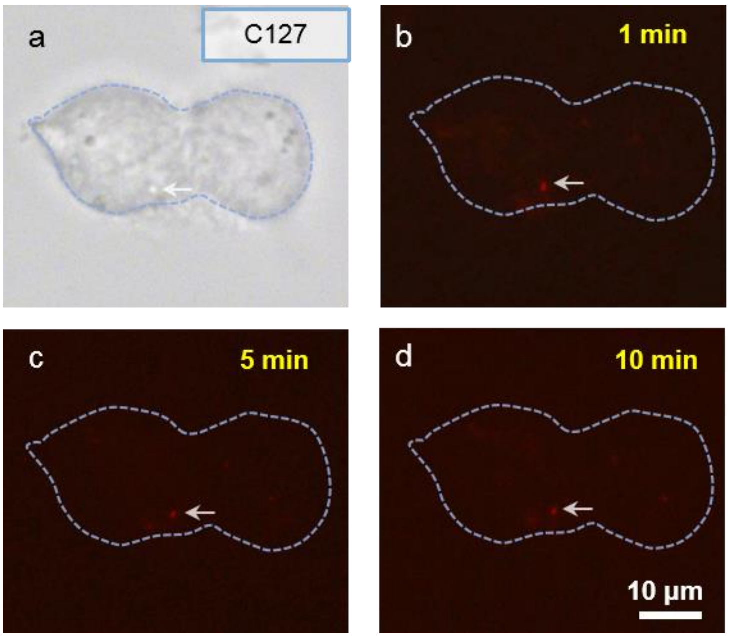

Figure S13. a) Confocal microscope image of an aggregated FND microsphere in a C127 cell. b-d) Fluorescence images of the aggregated FND microspheres in the C127 cell at 1 min (b), 5 min (c) and 10 min (d) after tuning off the trapping laser.

S4.2. FND aggregation in different cell types

To investigate the effect of different cell types on the FND aggregate formation at the same concentration of FND solution (Figure S14a). It can be seen that attributed to the stronger endocytosis ability of the Ana-1 cells (Phagocyte),[S5] more FNDs were endocytosed and aggregated in the Ana-1 cells than those in other cells (Figure S14b). Note that the aggregates

in the HeLa cell in Figure S14b2 was smaller than that in Figure 3c3 in the manuscript because the laser irradiation duration was 1 min less. At the same FND concentration, the amount of the FNDs endocytosed by the cells was much less than that dispersed in deionized water, so the time required to stabilize the FND aggregates became at least 5 times longer in the cells (Figure S14c). Because the endocytosed FNDs can be transported around the nucleus by the myosin so that it took less time to assemble the aggregates around the nuclei of the cells (red pillars in Figure S14c). Due to the limited endocytosis capacity of the cells, the diameters of the FND aggregates were smaller than that formed in deionized water under the same trapping laser power and duration of irradiation (Figure S14d). In addition, the larger size of the FND aggregates in water resulted a stronger final fluorescence intensity than those in the cells (Figure S14e).

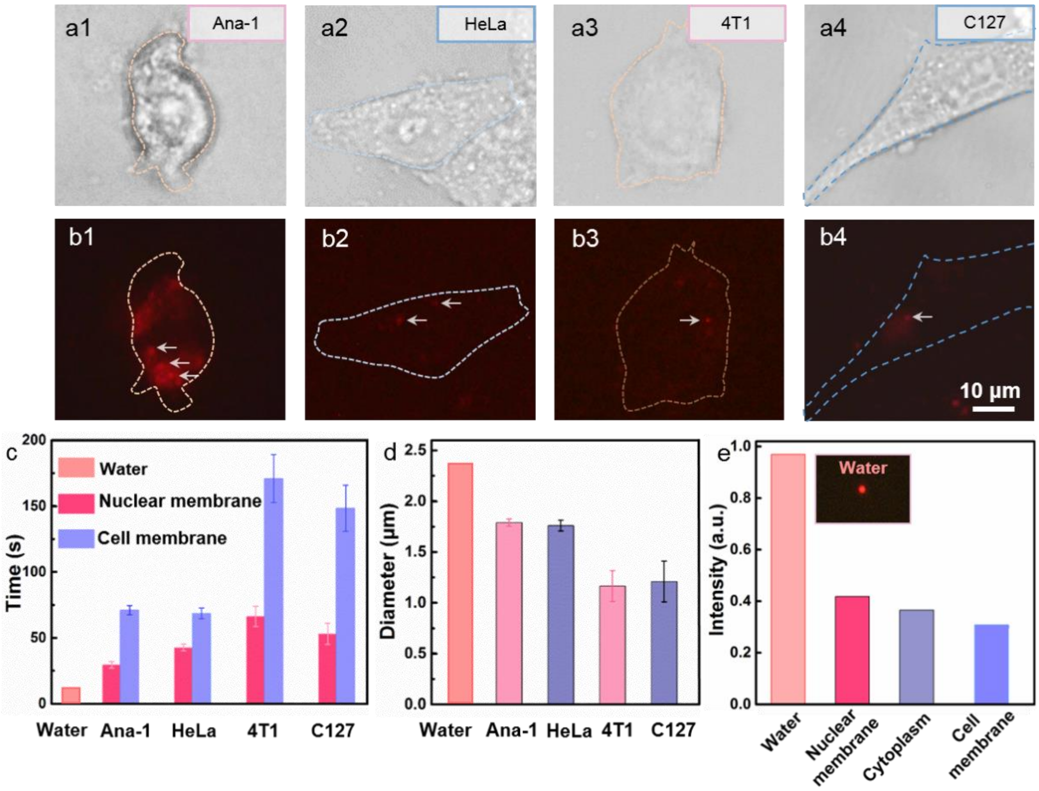

Figure S14. a) Confocal microscope images of Ana-1(a1), HeLa (a2), 4T1 (a3) and C127 cell (a4). The dashed lines represent the outlines of the cells. b) Fluorescence images of the aggregated FND microspheres in the Ana (b1), HeLa (b2), 4T1 (b3) and C127 cell (b4). Red: the aggregated FND microspheres excited by 546-nm laser, white arrows pointed to the aggregated FND microspheres. c) Histogram of the time required to aggregated FNDs in the

cells and DI water. d) Histogram of FND aggregate diameters in the four types of cells. e) Normalized Fluorescence intensity of the aggregated FND microspheres gathered in water, close to the cell nuclear and membrane, respectively.

S4.3. Moving FND aggregates within the cell

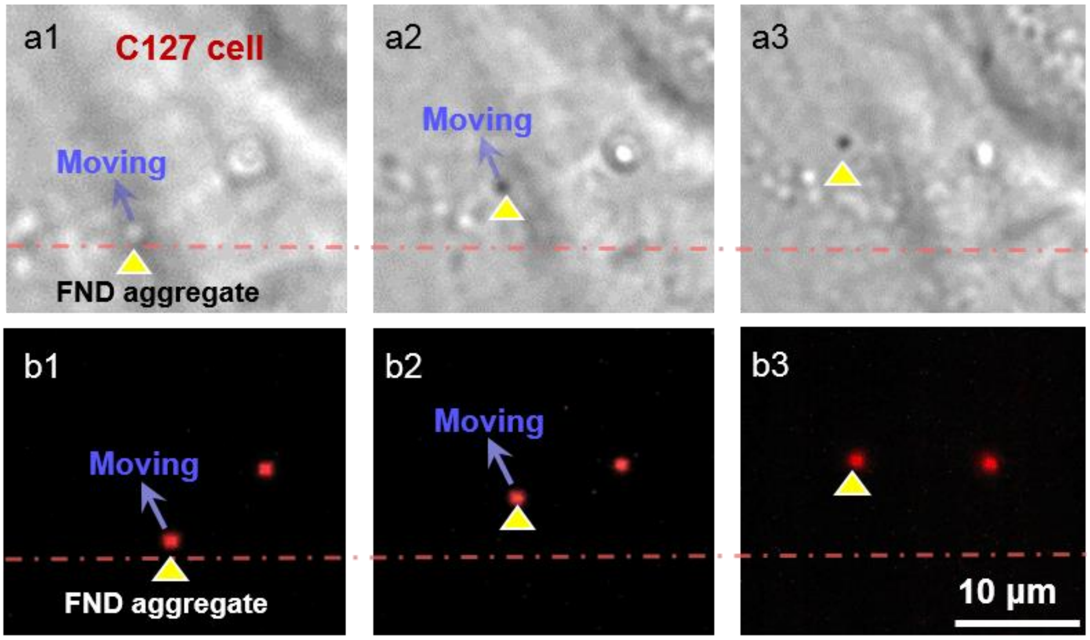

Figure S15. a) Optical microscope images of moving an FND microsphere within a living C127 cell by the SOTS. b) Fluorescence images corresponding to (a).

S4.4. Cell viability

To investigate the possible cytotoxic effects caused by the trapping laser or the FND aggregates, an experiment was carried out to indicate the cell viability after trapping the FNDs by the optical tweezers in living cells (Figure S16). In each group of the experiments, the effect was investigated for different trapping powers (Figure S16a), irradiation durations (Figure S16b) and amounts of FND aggerates (Figure S16c). The cell viability was indicated by staining the cells with the cell permeable nucleus counterstain (Hoechst 33342) after aggregating the FNDs in the cells. The results show that for the cells treated with an irradiation of 3 min, the survival rates were higher than 80% at trapping powers lower than 150 mW (Figure S16d). With a power fixed at 150 mW, the survival rates reached above 80%

within irradiation duration of 5 min (Figure S16e). In addition, the endocytosis of the FNDs has little effect on the cell survival because of the good biocompatibility (Figure S16f).

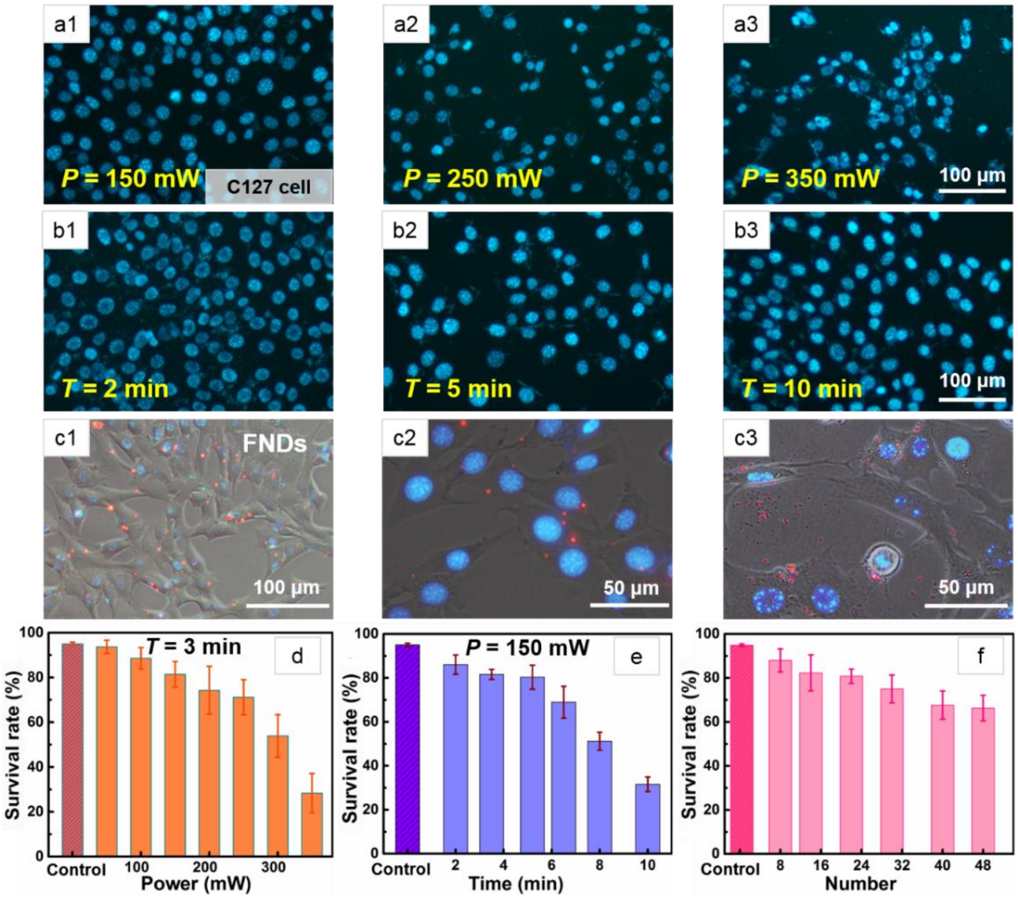

Figure S16. a) Fluorescence images of the living C127 cells (labeled with Hoechst 33342) irradiated for 3 min with laser powers of 150 (a1), 250 (a2) and 350 mW (a3). b) Fluorescence images of the cell irradiated for 2 (b1), 5 (b2) and 10 min (b3) with a laser power of 150 mW. c) Merged images of cells containing aggregated FND microspheres (c1), enlarged views of the living (c2) and dead cells (c3). d-f) Histograms of the cell survival rates at different trapping laser powers (d), trapping durations (e) and amounts of the FND aggregates (f).

S4.5. Microsphere precision in the cell

The precisions of the microsphere in the *x* and *y* directions can be experimentally measured, as shown in Figure S17. With a trapping laser power of 60 mW (the minimum power required to manipulate the aggregates in the cell), the vibration of an aggregated FND microsphere (2 μm) in the *x* and *y* directions was recorded (Figure S17a), indicating that the precisions of the *x* and *y* directions were 60 and 70 nm, respectively. In general, the precision in the *z* direction

is twice of that in the *x* or *y* direction. By increasing the trapping laser power, the precisions in three dimensions can be improved (Figure S17b). For the case in Figure 5, the trapping laser power was 150 mW, and the precisions of the *x*, *y*, and *z* were 27, 28, 56 nm, respectively. The trapping laser was applied near the outline of the cell membrane determined by the bright-field observation. After 3 minutes of laser irradiation, the FND aggregates were formed near the membrane and excited with 546-nm laser in dark field.

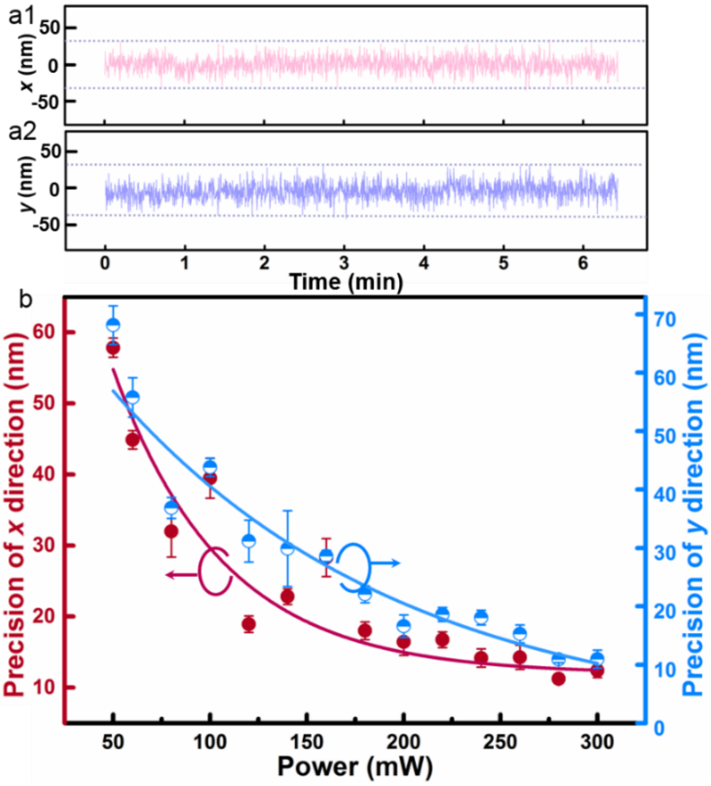

Figure S17. a) Recorded vibration of an aggregated FND microsphere (2 μm) in the *x* and *y* directions. b) Precisions as functions of the trapping laser power.

S4.6. ZPL measurement at 40 °C

At each temperature between 20 and 60 °C, four aggregated FND microspheres particles were used for the ZPL measurement. For example, the ZPLs of the four aggregates were measured at the temperature of 40°C (Figure S18), and the wavelength error was 0.06 nm.

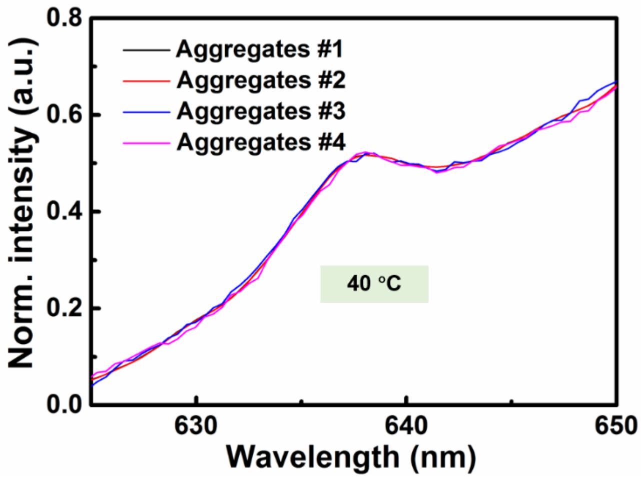

Figure S18. Fluorescence spectra of four aggregated FND microspheres in water at 40 °C.

References

[S1] L. M. Maestro, E. M. Rodríguez, F. S. Rodríguez, M. I. D. la Cruz, A. Juarranz, R. Naccache, J. G. Solé, *Nano Lett.* **2010**, *10*, 5109.

[S2] Y. Li, X. Liu, X. Yang, H. Lei, Y. Zhang, B. Li, *ACS Nano* **2017**, *11*, 10672.

[S3] G. Kucsko, P. C. Maurer, N. Y. Yao, M. I. C. H. A. E. L. Kubo, H. J. Noh, P. K. Lo, M. D. Lukin, *Nature* **2013**, *500*, 54.

[S4] Y. S. Al-Degs, M. I. El-Barghouthi, A. H. El-Sheikh, G. M. Walker, *Dyes Pigm*. **2008**, *77*, 16.

[S5] X. Cheng, X. Tian, A. Wu, J. Li, J. Tian, Y. Chong, C. Ge, *ACS Appl. Mater. Inter.* **2015**, *7*, 20568.

Supporting videos:

Video S1: FNDs aggregation process.

Video S2: Pattering of aggregated FNDs.

Video S3: Intracellular aggregation and patterning.
